# A Characterization of Maximally Entangled Two-Qubit States

**DOI:** 10.3390/e24020247

**Published:** 2022-02-07

**Authors:** Junjun Duan, Lin Zhang, Quan Qian, Shao-Ming Fei

**Affiliations:** 1 School of Sciences, Hangzhou Dianzi University, Hangzhou 310018, China; epochduan@163.com (J.D.); cauchyss@163.com (Q.Q.); 2 Max Planck Institute for Mathematics in the Sciences, 04103 Leipzig, Germany; feishm@cnu.edu.cn; 3 School of Mathematical Sciences, Capital Normal University, Beijing 100048, China

**Keywords:** maximally entangled state, positive partial transpose, moment

## Abstract

As already known by Rana’s result, all eigenvalues of any partial-transposed bipartite state fall within the closed interval $\left[-\frac{1}{2},1\right]$. In this note, we study a family of bipartite quantum states where the minimal eigenvalues of partial-transposed states are $-\frac{1}{2}$. For a two-qubit system, we find that the minimal eigenvalue of its partial-transposed state is $-\frac{1}{2}$ if and only if such a two-qubit state is maximally entangled. However this result does not hold in general for a two-qudit system when the dimensions of the underlying space are larger than two.

## 1. Introduction

Let $\rho_{AB}$ be a quantum state in a bipartite quantum system $H_{A}⊗H_{B}$ such that the positive partial transpose (PPT) criterion indicates that, for any separable state $\rho_{AB}$, it must hold $\rho_{AB}^{T_{A}}⩾0$, where ${}^{T_{A}}$ denotes the partial transpose on subsystem *A*. This PPT condition was first proposed by Peres [1]. Such a condition is not only a necessary one but also a sufficient one for separability in a qubit–qubit, qubit–qutrit or a qutrit–qubit system [2]. The PPT condition can also be verified from the moments of the randomized measurements [3,4,5].

Recently, Yu et al. [6] found that the PPT condition can be studied by considering the so-called partial transpose moments (PT-moments)

$$\begin{array}{c} p_{k}:={\mathrm{Tr}}_{}\left({\left[\rho_{AB}^{T_{A}}\right]}^{k}\right). \end{array}$$

In fact, these quantities can be efficiently measured in experiments [4,5]. To see the basic idea behind the PT-moments-based entanglement detection, suppose that we know all the PT-moments $p^{\left(d\right)}=\left(p_{1},\ldots ,p_{d}\right)$, where $d=d_{A}d_{B}$ is the dimension of the global system $H_{A}⊗H_{B}$, where $d_{A/B}=\dim\left(H_{A/B}\right)$. We call $p^{\left(d\right)}$ the PT-moment vector of the state $\rho_{AB}$. All the eigenvalues of $\rho_{AB}^{T_{A}}$ are denoted by $\left(x_{1},\ldots ,x_{d}\right)$. As is already known, $\left(x_{1},\ldots ,x_{d}\right)$ completely determines the elementary symmetric polynomials $\left(e_{1},\ldots ,e_{d}\right)$, where $e_{1}=\sum_{k=1}^{d}x_{k}$, $e_{2}=\sum_{1⩽i<j⩽d}x_{i}x_{j},\ldots$, and $e_{d}=\prod_{k=1}^{d}x_{k}$; and conversely $\left(e_{1},\ldots ,e_{d}\right)$ can determine $\left(x_{1},\ldots ,x_{d}\right)$ when ignoring their order. In fact, $\left(e_{1},\ldots ,e_{d}\right)$ and $\left(p_{1},\ldots ,p_{d}\right)$, where $p_{k}$’s are the power sum of $x_{i}$’s, necessarily identify each other via the following relationship between $e_{k}$ and $p_{k}$ [7]:

$$\begin{array}{c} p_{k}=\left|\begin{array}{ccccc} e_{1} & 1 & 0 & \cdots & 0\\ 2e_{2} & e_{1} & 1 & \cdots & 0\\ \vdots & \vdots & \vdots & \ddots & \vdots \\ \left(k-1\right)e_{k-1} & e_{k-2} & e_{k-3} & \cdots & 1\\ ke_{k} & e_{k-1} & e_{k-2} & \cdots & e_{1} \end{array}\right|\;\left(k⩾1\right) \end{array}$$

and

$$\begin{array}{c} e_{k}=\frac{1}{k!}\left|\begin{array}{ccccc} p_{1} & 1 & 0 & \cdots & 0\\ p_{2} & p_{1} & 2 & \cdots & 0\\ \vdots & \vdots & \vdots & \ddots & \vdots \\ p_{k-1} & p_{k-2} & p_{k-3} & \cdots & k-1\\ p_{k} & p_{k-1} & p_{k-2} & \cdots & p_{1} \end{array}\right|\;\left(k⩾1\right). \end{array}$$

Therefore $\left(p_{1},\ldots ,p_{d}\right)$ determines $\left(x_{1},\ldots ,x_{d}\right)$ up to their order. Then, all eigenvalues of the partial-transposed state $\rho_{AB}^{T_{A}}$ can be directly obtained. Based on the above observation, the PPT criterion can be verified immediately. For convenience, we always assume that $p_{1}=1$. In addition, $p_{2}$ is just the purity due to the fact that ${\mathrm{Tr}}_{}\left({\left[\rho_{AB}^{T_{A}}\right]}^{2}\right)={\mathrm{Tr}}_{}\left(\rho_{AB}^{2}\right)$. In [6], the authors studied the following problem.

**PT-Moment problem:** Given the PT-moments of order *n*, is there a separable state compatible with the data? In more technical language, given the PT-moment vector

$$\begin{array}{c} p^{\left(n\right)}=\left(p_{1},\ldots ,p_{n}\right), \end{array}$$

is there a separable quantum state $\rho_{AB}$ such that

$$\begin{array}{c} p_{k}={\mathrm{Tr}}_{}\left({\left[\rho_{AB}^{T_{A}}\right]}^{k}\right),\;k=1,\ldots ,n? \end{array}$$

It is natural to consider the detection of entanglement in $\rho_{AB}$ from a few of the PT-moments due to the difficulty in measuring all the PT-moments, such as in [6]. Note that the partial-transposed state $\rho_{AB}^{T_{A}}$, for $\rho_{AB}\in D\left(C^{m}⊗C^{n}\right)$, which is the set of all bipartite quantum states acting on $C^{m}⊗C^{n}$, cannot have more than (m−1)(n−1) negative eigenvalues and all eigenvalues of $\rho_{AB}^{T_{A}}$ fall within $\left[-\frac{1}{2},1\right]$ [8]. Using the second PT-moment to bound the third one is an interesting question. Moreover, we find that this method can be used to get a characterization of maximally entangled two-qubit states, that is, a two-qubit state is maximally entangled if and only if the minimal eigenvalue of its partial-transposed state is $-\frac{1}{2}$. This is also equivalent to the condition that $p^{\left(4\right)}=\left(1,1,\frac{1}{4},\frac{1}{4}\right)$, the PT-moment vector of the two-qubit state $\rho_{AB}$. This amounts to giving the criterion of maximal entanglement to the states using the PT-moment vector, the components of which are measurable quantities.

## 2. Main Result

In this section, we essentially ask: Is $\rho_{AB}$ maximally entangled if $\rho_{AB}\in D\left(C^{2}⊗C^{2}\right)$ and the minimal eigenvalue of its partial-transposed state $\rho_{AB}^{T_{A}}$ is $\lambda_{\min}\left(\rho_{AB}^{T_{A}}\right)=-\frac{1}{2}$? We give a positive answer to this question in our main result, i.e., Theorem 1. To that end, we obtained the proof through a series of propositions.

**Proposition** **1.** *Let $\rho_{AB},\sigma_{AB},\tau_{AB}\in D\left(C^{m}⊗C^{n}\right)$, where $\rho_{AB}=t\sigma_{AB}+\left(1-t\right)\tau_{AB}$ for some t∈(0,1). If $\lambda_{\min}\left(\rho_{AB}^{T_{A}}\right)=-\frac{1}{2}$, i.e., the minimal eigenvalue of $\rho_{AB}^{T_{A}}$, then $\lambda_{\min}\left(\sigma_{AB}^{T_{A}}\right)=\lambda_{\min}\left(\tau_{AB}^{T_{A}}\right)=-\frac{1}{2}$.*

**Proof.**  Using the main result in [8], we see that, for any bipartite state $\varrho_{AB}\in D\left(C^{m}⊗C^{n}\right)$, we have

$$\begin{array}{c} \lambda_{\min}\left(\varrho_{AB}^{T_{A}}\right)\in \left[-\frac{1}{2},1\right]. \end{array}$$

Thus,

$$\begin{array}{c} \lambda_{\min}\left(\sigma_{AB}^{T_{A}}\right)⩾-\frac{1}{2},\;\lambda_{\min}\left(\tau_{AB}^{T_{A}}\right)⩾-\frac{1}{2}. \end{array}$$

As is already known, there exists a pure state $|\psi_{0}\rangle \in C^{m}⊗C^{n}$, corresponding to the minimal eigenvalue $\lambda_{\min}\left(\rho_{AB}^{T_{A}}\right)$, such that

$$\begin{array}{ccc} -\frac{1}{2} & = & \lambda_{\min}\left(\rho_{AB}^{T_{A}}\right)=\langle \psi_{0}\left|\rho_{AB}^{T_{A}}\right|\psi_{0}\rangle =t\langle \psi_{0}\left|\sigma_{AB}^{T_{A}}\right|\psi_{0}\rangle +\left(1-t\right)\langle \psi_{0}\left|\tau_{AB}^{T_{A}}\right|\psi_{0}\rangle \\ & ⩾ & t\lambda_{\min}\left(\sigma_{AB}^{T_{A}}\right)+\left(1-t\right)\lambda_{\min}\left(\tau_{AB}^{T_{A}}\right)⩾-\frac{1}{2}. \end{array}$$

We must have that $\lambda_{\min}\left(\sigma_{AB}^{T_{A}}\right)=\langle \psi_{0}\left|\sigma_{AB}^{T_{A}}\right|\psi_{0}\rangle =\lambda_{\min}\left(\tau_{AB}^{T_{A}}\right)=\langle \psi_{0}\left|\tau_{AB}^{T_{A}}\right|\psi_{0}\rangle =-\frac{1}{2}$. □

**Corollary** **1.** *Suppose $\rho_{AB}\in D\left(C^{m}⊗C^{n}\right)$ has the pure state decomposition: $\rho_{AB}=\sum_{k}\lambda_{k}|\psi_{k}\rangle \;\langle \psi_{k}|$, where $\lambda_{k}>0$ for all indices k. If $\lambda_{\min}\left(\rho_{AB}^{T_{A}}\right)=-\frac{1}{2}$, then*

$$\begin{array}{c} \lambda_{\min}\left(\psi_{k}^{T_{A}}\right)=-\frac{1}{2}. \end{array}$$

*Here $\psi_{k}:=|\psi_{k}\rangle \;\langle \psi_{k}|$.*

Recall that there is a correspondence between the set $L\left(Y,X\right)$ of all linear operators from a finite-dimensional Hilbert space Y to another finite-dimensional Hilbert space X. It can be explained immediately. Denote by X⊗Y the tensor space of X and Y. Let the orthonormal bases of X and Y be $\left\{|i\rangle :i=1,\ldots ,\dim(X)\right\}$ and $\left\{|j\rangle :j=1,\ldots ,\dim(Y)\right\}$, respectively. The mentioned correspondence between $L\left(Y,X\right)$ and X⊗Y is defined by the linear mapping $\mathrm{vec}:L\left(Y,X\right)\to X⊗Y$ via vec(|i〉〈j|)=|ij〉 for all i,j [9].

Let $|\psi \rangle \in C^{d}⊗C^{d}$ be a bipartite pure state. Then there is an d×d complex matrix X such that |ψ〉=vec(X). By singular value decomposition (SVD), there are two unitary matrices U,V∈U(d) such that $X=U\Sigma V^{†}$, where $\Sigma =\mathrm{diag}(\sigma_{1},\ldots ,\sigma_{r},\ldots ,\sigma_{d})$ for $\sigma_{1}⩾\cdots ⩾\sigma_{d}⩾0$ and r=rank(X)⩽d. Note that $\sum_{j=1}^{d}\sigma_{j}^{2}=1$. Then

$$\begin{array}{c} \left|\psi \rangle \;\langle \psi \right|=U⊗\bar{V}\mathrm{vec}\left(\Sigma \right)\mathrm{vec}{\left(\Sigma \right)}^{†}{\left(U⊗\bar{V}\right)}^{†} \end{array}$$

implying that

$$\begin{array}{c} {\left|\psi \rangle \;\langle \psi \right|}^{T_{A}}=\bar{U}⊗\bar{V}{\left(\mathrm{vec}\left(\Sigma \right)\mathrm{vec}{\left(\Sigma \right)}^{†}\right)}^{T_{A}}{\left(\bar{U}⊗\bar{V}\right)}^{†}. \end{array}$$

Due to the fact that $\Sigma =\sum_{i=1}^{d}\sigma_{i}\left|i\rangle \;\langle i\right|$, we see that

$$\begin{array}{c} \mathrm{vec}\left(\Sigma \right)\mathrm{vec}{\left(\Sigma \right)}^{†}=\sum_{i,j=1}^{d}\sigma_{i}\sigma_{j}|ij\rangle \langle ij|,\;{\left(\mathrm{vec}\left(\Sigma \right)\mathrm{vec}{\left(\Sigma \right)}^{†}\right)}^{T_{A}}=\sum_{i,j=1}^{d}\sigma_{i}\sigma_{j}\left|ji\rangle \langle ij\right| \end{array}$$

**Proposition** **2.** *All eigenvalues of ${\left|\psi \rangle \;\langle \psi \right|}^{T_{A}}$ is given by $\left\{\sigma_{1}^{2},\ldots ,\sigma_{d}^{2};\pm \sigma_{i}\sigma_{j}\;\left(1⩽i<j⩽d\right)\right\}$.*

**Proof.**  Let $F={\left(\mathrm{vec}\left(\Sigma \right)\mathrm{vec}{\left(\Sigma \right)}^{†}\right)}^{T_{A}}=\sum_{i,j=1}^{d}\sigma_{i}\sigma_{j}\left|ji\rangle \langle ij\right|$. Then

$$\begin{array}{ccc} FF^{†} & = & \left(\sum_{i,j=1}^{d}\sigma_{i}\sigma_{j}\left|ji\rangle \langle ij\right|\right){\left(\sum_{k,l=1}^{d}\sigma_{k}\sigma_{l}\left|lk\rangle \langle kl\right|\right)}^{†}=\sum_{i,j,k,l=1}^{d}\sigma_{i}\sigma_{j}\sigma_{k}\sigma_{l}\left|ji\rangle \langle ij\right|\cdot \left|kl\rangle \langle lk\right|\\ & = & \sum_{i,j,k,l=1}^{d}\delta_{ik}\delta_{jl}\sigma_{i}\sigma_{j}\sigma_{k}\sigma_{l}\left|ji\rangle \langle lk\right|=\sum_{i,j=1}^{d}{\left(\sigma_{i}\sigma_{j}\right)}^{2}|ji\rangle \langle ji|; \end{array}$$

that is, $\left|F\right|=\sqrt{FF^{†}}=\sum_{i,j=1}^{d}\sigma_{i}\sigma_{j}\left|ji\rangle \langle ji\right|$. Note that

$$\begin{array}{c} {\mathrm{Tr}}_{}\left(F\right)=\sum_{i=1}^{d}\sigma_{i}^{2},\;{\mathrm{Tr}}_{}\left(|F|\right)=\sum_{i,j=1}^{d}\sigma_{i}\sigma_{j}. \end{array}$$

By using the Jordan decomposition $F=F_{+}-F_{-}$, where $F_{\pm }^{†}=F_{\pm }⩾0$ and $F_{+}F_{-}=0=F_{-}F_{+}$. Then

$$\begin{array}{c} {\mathrm{Tr}}_{}\left(F_{+}\right)-{\mathrm{Tr}}_{}\left(F_{-}\right)=\sum_{i=1}^{d}\sigma_{i}^{2},\;{\mathrm{Tr}}_{}\left(F_{+}\right)+{\mathrm{Tr}}_{}\left(F_{-}\right)=\sum_{i,j=1}^{d}\sigma_{i}\sigma_{j}, \end{array}$$

and

$$\begin{array}{c} {\mathrm{Tr}}_{}\left(F_{+}\right)=\sum_{i=1}^{d}\sigma_{i}^{2}+\sum_{i<j}\sigma_{i}\sigma_{j},\;{\mathrm{Tr}}_{}\left(F_{-}\right)=\sum_{i<j}\sigma_{i}\sigma_{j}. \end{array}$$

Therefore, all eigenvalues of F are given by $\left\{\sigma_{1}^{2},\ldots ,\sigma_{d}^{2};\pm \sigma_{i}\sigma_{j}\;\left(1⩽i<j⩽d\right)\right\}$. □

Thus, we need to characterize those bipartite pure states that have the minimal eigenvalue of its partial-transposed state, $-\frac{1}{2}$.

**Proposition** **3.** *If $|\psi \rangle \in C^{2}⊗C^{2}$ is a pure state, then $\lambda_{\min}\left(\psi^{T_{A}}\right)=-\frac{1}{2}$, where ψ≡|ψ〉〈ψ|, if and only if |ψ〉 is a maximally entangled state, i.e., |ψ〉 is proportional to the locally unitarily rotation of the vector $\mathrm{vec}(I_{2})$. Here I is the identity operator.*

**Proof.**  Let $|\psi \rangle \in C^{2}⊗C^{2}$. Suppose $x=\left(x_{1},x_{2},x_{3},x_{4}\right)\in R^{4}$ is the eigenvalues of $\psi^{T_{A}}$ with $1⩾x_{1}⩾x_{2}⩾x_{3}⩾x_{4}⩾-\frac{1}{2}$ [8]. Clearly $x_{4}=\lambda_{\min}\left(\psi^{T_{A}}\right)$.

Now let $x_{4}=-\frac{1}{2}$. Again, we see that $x_{3}⩾0$ by Rana’s result. Let $p_{k}={\mathrm{Tr}}_{}\left({\left[\psi^{T_{A}}\right]}^{k}\right)$, where k=1,2,…. It is easy to see that $p_{1}=1,p_{2}=1$. Then

$$\begin{array}{c} \left\{\begin{array}{c} 1=x_{1}+x_{2}+x_{3}+\left(-\frac{1}{2}\right)\\ 1=x_{1}^{2}+x_{2}^{2}+x_{3}^{2}+{\left(-\frac{1}{2}\right)}^{2}. \end{array}\right. \end{array}$$

Due to the constraint $1⩾x_{1}⩾x_{2}⩾x_{3}⩾0$, the above system of equations has a unique solution: $x_{1}=x_{2}=x_{3}=\frac{1}{2}$.

We have now proved that if $\lambda_{\min}\left(\psi^{T_{A}}\right)=-\frac{1}{2}$ for some pure state $|\psi \rangle \in C^{2}⊗C^{2}$, then all eigenvalues of $\psi^{T_{A}}$ are $\left\{\frac{1}{2},\frac{1}{2},\frac{1}{2},-\frac{1}{2}\right\}$. In fact, if $\lambda_{\min}\left(\rho_{AB}^{T_{A}}\right)=-\frac{1}{2}$ for some state $\rho_{AB}\in D\left(C^{2}⊗C^{2}\right)$, then all eigenvalues of $\rho_{AB}^{T_{A}}$ is $\left\{\frac{1}{2},\frac{1}{2},\frac{1}{2},-\frac{1}{2}\right\}$.

For a pure state $|\psi \rangle \in C^{2}⊗C^{2}$, there exists a 2×2 complex matrix A such that |ψ〉=vec(A). By SVD of A, we get that $A=UDV^{†}$ where $D=\mathrm{diag}(s_{0},s_{1})$ with $s_{0}⩾s_{1}⩾0$ and U,V∈U(2). Then

$$\begin{array}{ccc} \left|\psi \rangle \;\langle \psi \right| & = & U⊗\bar{V}\mathrm{vec}\left(D\right)\mathrm{vec}{\left(D\right)}^{†}{\left(U⊗\bar{V}\right)}^{†}\\ & = & U⊗\bar{V}\mathrm{vec}\left(D\right)\mathrm{vec}{\left(D\right)}^{†}U^{†}⊗V^{T}, \end{array}$$

where

$$\begin{array}{c} \mathrm{vec}\left(D\right)\mathrm{vec}{\left(D\right)}^{†}=\mathrm{vec}\left(\sum_{i}s_{i}\left|i\rangle \;\langle i\right|\right)\mathrm{vec}{\left(\sum_{j}s_{j}\left|j\rangle \;\langle j\right|\right)}^{†}=\sum_{i,j=0}^{1}s_{i}s_{j}|ii\rangle \langle jj|. \end{array}$$

Next, we established the equations concerning $\left(s_{0},s_{1}\right)$. The first one is $s_{0}^{2}+s_{1}^{2}=1$ due to the fact that ${\mathrm{Tr}}_{}\left(D^{2}\right)=\langle \psi ,\psi \rangle =1$. The second one is

$$\begin{array}{ccc} {\left|\psi \rangle \;\langle \psi \right|}^{T_{A}} & = & \sum_{i,j=0}^{1}s_{i}s_{j}(U|i\rangle {\langle j|U^{†})}^{T}⊗\bar{V}\left|i\rangle \langle j\right|V^{T}\\ & = & \sum_{i,j=0}^{1}s_{i}s_{j}\bar{U}\left|j\rangle \langle i\right|U^{T}⊗\bar{V}\left|i\rangle \langle j\right|V^{T}\\ & = & \left(\bar{U}⊗\bar{V}\right)\sum_{i,j=0}^{1}s_{i}s_{j}\left|j\rangle \langle i\right|⊗\left|i\rangle \langle j\right|{\left(\bar{U}⊗\bar{V}\right)}^{†}\\ & = & \left(\bar{U}⊗\bar{V}\right)\sum_{i,j=0}^{1}s_{i}s_{j}\left|ji\rangle \langle ij\right|{\left(\bar{U}⊗\bar{V}\right)}^{†}. \end{array}$$

Now both ${\left|\psi \rangle \;\langle \psi \right|}^{T_{A}}$ and $\sum_{i,j=0}^{1}s_{i}s_{j}\left|ji\rangle \langle ij\right|$ have the same eigenvalues. That is, all eigenvalues of

$$\begin{array}{c} \sum_{i,j=0}^{1}s_{i}s_{j}\left|ji\rangle \langle ij\right|=\left(\begin{array}{cccc} s_{1}^{2} & 0 & 0 & 0\\ 0 & 0 & s_{1}s_{2} & 0\\ 0 & s_{1}s_{2} & 0 & 0\\ 0 & 0 & 0 & s_{2}^{2} \end{array}\right) \end{array}$$

are $\left\{s_{0}^{2},s_{1}^{2},s_{0}s_{1},-s_{0}s_{1}\right\}=\left\{\frac{1}{2},\frac{1}{2},\frac{1}{2},-\frac{1}{2}\right\}$. This implies that

$$\begin{array}{c} s_{0}^{2}=s_{1}^{2}=s_{0}s_{1}=\frac{1}{2}. \end{array}$$

This unique solution is given by $\left(s_{0},s_{1}\right)=\left(\frac{1}{\sqrt{2}},\frac{1}{\sqrt{2}}\right)$. Therefore, $A=\frac{1}{\sqrt{2}}UV^{†}$. Then

$$\begin{array}{c} |\psi \rangle =\mathrm{vec}\left(A\right)=\frac{1}{\sqrt{2}}\mathrm{vec}\left(UV^{†}\right)=\frac{1}{\sqrt{2}}U⊗\bar{V}\mathrm{vec}\left(I_{2}\right). \end{array}$$

We have proven that |ψ〉 is a maximally entangled state. Conversely, if |ψ〉 is a maximally entangled state, then the minimal eigenvalue of its partial-transposed state is apparently $-\frac{1}{2}$ [10,11]. □

For the partial-transposed maximally entangled states in $C^{n}⊗C^{n}$, the eigenvalues must be $\pm \frac{1}{n}$ where the multiplicities of $\frac{1}{n}$ and $-\frac{1}{n}$ are $\frac{n(n+1)}{2}$ and $\frac{n(n-1)}{2}$, respectively. Thus its PT-moment vector is given by

$$\begin{array}{c} p^{\left(n^{2}\right)}=\left(p_{1},\ldots ,p_{n^{2}}\right),\;p_{k}=\frac{\left(n+1\right)+\left(n-1\right){\left(-1\right)}^{k}}{2n^{k-1}}. \end{array}$$

In particular, for the case where n=2, the PT-moment vector $p^{\left(4\right)}=\left(1,1,\frac{1}{4},\frac{1}{4}\right)$.

**Theorem** **1.** *Let $\rho_{AB}\in D\left(C^{2}⊗C^{2}\right)$ be a quantum state, then the following statements are equivalent:*

- *the PT-moment vector of $\rho_{AB}$ is $p^{\left(4\right)}=\left(1,1,\frac{1}{4},\frac{1}{4}\right)$.*
- *$\rho_{AB}$ must be maximally entangled.*

**Proof.**  For the implication (ii) ⟹ (i), the proof is trivial. Next, we show that (i) implies (ii). Given (i), let $\left(x_{1},x_{2},x_{3},x_{4}\right)$, where $x_{1}⩾x_{2}⩾x_{3}⩾x_{4}$, be eigenvalues of the partial-transposed state $\rho_{AB}^{T_{A}}$, then Rana’s result [8] means that $1⩾x_{1}⩾x_{2}⩾x_{3}⩾\left\{\begin{array}{c} 0\\ x_{4}⩾-\frac{1}{2} \end{array}\right.$. By the given PT-moment vector, we see that

$$\begin{array}{c} \left\{\begin{array}{c} p_{1}=x_{1}+x_{2}+x_{3}+x_{4}=1\\ p_{2}=x_{1}^{2}+x_{2}^{2}+x_{3}^{2}+x_{4}^{2}=1\\ p_{3}=x_{1}^{3}+x_{2}^{3}+x_{3}^{3}+x_{4}^{3}=\frac{1}{4}\\ p_{4}=x_{1}^{4}+x_{2}^{4}+x_{3}^{4}+x_{4}^{4}=\frac{1}{4} \end{array}\right. \end{array}$$

In fact, note that

$$\begin{array}{c} e_{k}=\frac{1}{k!}\left|\begin{array}{ccccc} p_{1} & 1 & 0 & \cdots & 0\\ p_{2} & p_{1} & 2 & \cdots & 0\\ \vdots & \vdots & \vdots & \ddots & \vdots \\ p_{k-1} & p_{k-2} & p_{k-3} & \cdots & k-1\\ p_{k} & p_{k-1} & p_{k-2} & \cdots & p_{1} \end{array}\right|\;\left(k⩾1\right), \end{array}$$

and we see that $e_{1}=1,e_{2}=0,e_{3}=-\frac{1}{4},e_{4}=-\frac{1}{16}$. The characteristic polynomial of $\rho_{AB}^{T_{A}}$ is given by

$$\begin{array}{ccc} f(x) & = & x^{4}-e_{1}x^{3}+e_{2}x^{2}-e_{3}x+e_{4}=x^{4}-x^{3}+\frac{1}{4}x-\frac{1}{16}\\ & = & \frac{1}{16}{\left(2x-1\right)}^{3}\left(2x+1\right). \end{array}$$

Solving this system of equations via f(x)=0, we get that $x_{1}=x_{2}=x_{3}=\frac{1}{2}$ and $x_{4}=\lambda_{\min}\left(\rho_{AB}^{T_{A}}\right)=-\frac{1}{2}$. Next, if $F=\sum_{i,j=0}^{1}\left|ji\rangle \langle ij\right|$, then F|ij〉=|ji〉 and $F^{T_{A}}=\mathrm{vec}\left(I\right)\mathrm{vec}{\left(I\right)}^{†}$. Note that $F\frac{|01\rangle -|10\rangle }{\sqrt{2}}=-\frac{|01\rangle -|10\rangle }{\sqrt{2}}$. Let $|x\rangle =\frac{|01\rangle -|10\rangle }{\sqrt{2}}$, and we get $\langle x\left|F\right|x\rangle =-1=\lambda_{\min}\left(F\right)$.

From the previous discussion, we see that in the pure state decomposition of $\rho_{AB}$: $\rho_{AB}=\sum_{k=0}^{N-1}\lambda_{k}|\psi_{k}\rangle \;\langle \psi_{k}|$, where $\lambda_{0}⩾\lambda_{1}⩾\cdots ⩾\lambda_{N-1}⩾0$, all pure state $|\psi_{k}\rangle$ must be maximally entangled state. Then there exist a pure state |u〉 such that

$$\begin{array}{c} \langle u\left|\psi_{k}^{T_{A}}\right|u\rangle =-\frac{1}{2}\;\left(k=0,1,\ldots ,N-1\right). \end{array}$$

There exist $U_{k},V_{k}\in U\left(2\right)$ such that

$$\begin{array}{c} |\psi_{k}\rangle =\frac{1}{\sqrt{2}}\mathrm{vec}\left(U_{k}V_{k}^{†}\right)=\frac{1}{\sqrt{2}}U_{k}V_{k}^{†}⊗I\mathrm{vec}\left(I\right)=\frac{1}{\sqrt{2}}W_{k}⊗I\mathrm{vec}\left(I\right), \end{array}$$

where $W_{k}=U_{k}V_{k}^{†}$, implying that $\psi_{k}^{T_{A}}=\frac{1}{2}\left({\bar{W}}_{k}⊗I\right)F{\left({\bar{W}}_{k}⊗I\right)}^{†}$. Now let $|u_{k}\rangle =W_{k}^{T}⊗I|u\rangle$. Then

$$\begin{array}{c} -\frac{1}{2}=\langle u\left|\psi_{k}^{T_{A}}\right|u\rangle =\frac{1}{2}\langle u_{k}\left|F\right|u_{k}\rangle \;\left(k=0,1,\ldots ,N-1\right). \end{array}$$

That is,

$$\begin{array}{c} \lambda_{\min}\left(F\right)=-1=\langle u_{k}\left|F\right|u_{k}\rangle \;\left(k=0,1,\ldots ,N-1\right). \end{array}$$

Because −1 is the simple eigenvalue of F, the eigenspace corresponding to −1 is just C|x〉. This indicates that all $|u_{k}\rangle =e^{i\theta_{k}}|x\rangle$ due to the normalization of $|u_{k}\rangle$. Furthermore

$$\begin{array}{c} |u\rangle =e^{i\theta_{k}}\left({\bar{W}}_{k}⊗I\right)|x\rangle \;\left(k=0,1,\ldots ,N-1\right). \end{array}$$

In fact, the phase factor $e^{i\theta_{k}}$ can be absorbed into the unitary matrix $W_{k}$. Without loss of generality, we assume that

$$\begin{array}{c} |u\rangle =\left({\bar{W}}_{k}⊗I\right)|x\rangle \;\left(k=0,1,\ldots ,N-1\right). \end{array}$$

Because there is a matrix X such that |x〉=vec(X), then

$$\begin{array}{c} X=\frac{1}{\sqrt{2}}(|0\rangle \langle 1|-|1\rangle \langle 0|)=\frac{1}{\sqrt{2}}\left(\begin{array}{cc} 0 & 1\\ -1 & 0 \end{array}\right). \end{array}$$

It is easily seen that X is invertible. We see that

$$\begin{array}{c} |u\rangle =\mathrm{vec}\left({\bar{W}}_{k}X\right)\;\left(k=0,1,\ldots ,N-1\right). \end{array}$$

implying that ${\bar{W}}_{0}X={\bar{W}}_{1}X=\cdots ={\bar{W}}_{N-1}X$, i.e., due to the fact that X is invertible, then $W_{0}=W_{1}=\cdots =W_{N-1}$, or

$$\begin{array}{c} U_{0}V_{0}^{†}=U_{1}V_{1}^{†}=\cdots =U_{N-1}V_{N-1}^{†}, \end{array}$$

implying that $\mathrm{vec}\left(U_{0}V_{0}^{†}\right)=\mathrm{vec}\left(U_{1}V_{1}^{†}\right)=\cdots =\mathrm{vec}\left(U_{N-1}V_{N-1}^{†}\right)$; that is, $|\psi_{0}\rangle =|\psi_{1}\rangle =\cdots =|\psi_{N-1}\rangle$. Therefore $\rho_{AB}=\sum_{k}\lambda_{k}|\psi_{k}\rangle \;\langle \psi_{k}\left|=\right|\psi_{0}\rangle \;\langle \psi_{0}|$ is a maximally entangled state. □

In fact, our main result, Theorem 1, tells us that the PT-moment vector of a two-qubit state $\rho_{AB}$ is $\left(1,1,\frac{1}{4},\frac{1}{4}\right)$ iff the minimal eigenvalue of its partial-transposed state $\rho_{AB}^{T_{A}}$ is $-\frac{1}{2}$ iff $\rho_{AB}$ is maximally entangled. Naturally, we would expect a similar relation between the magnitude of the lowest negative eigenvalue of the partial-transposed state and the maximally entangled states in higher-dimensional underlying spaces. However, the following result, Proposition 4, indicates that the minimal eigenvalue of the partial-transposed maximally entangled state would approach zero when the dimension of the underlying space becomes larger and larger. Indeed, after tedious computations and induction, we can draw the following conclusion:

**Proposition** **4.** *Let $\rho_{AB}\in D\left(C^{n}⊗C^{n}\right)$ be a quantum state. If the PT-moment vector of $\rho_{AB}$ is $p^{\left(n^{2}\right)}=\left(p_{1},\ldots ,p_{n^{2}}\right)$, where $p_{k}=\frac{\left(n+1\right)+\left(n-1\right){\left(-1\right)}^{k}}{2n^{k-1}}$. Then $\lambda_{\min}\left(\rho_{AB}^{T_{A}}\right)=-\frac{1}{n}$.*

**Proof.**  As an illustration, for a two-qutrit system $C^{3}⊗C^{3}$ as an example, we get $\left(e_{1},\ldots ,e_{9}\right)$ from $p^{\left(9\right)}=\left(p_{1},\ldots ,p_{9}\right)$, where $p_{1}=p_{2}=1,p_{3}=p_{4}=\frac{1}{9},p_{5}=p_{6}=\frac{1}{81},p_{7}=p_{8}=\frac{1}{729}$ and $p_{9}=\frac{1}{6561}$. That is,

$$\begin{array}{c} e_{1}=1,e_{2}=0,e_{3}=-\frac{8}{27},e_{4}=-\frac{2}{27},e_{5}=\frac{2}{81},\\ e_{6}=\frac{8}{729},e_{7}=0,e_{8}=-\frac{1}{2187},e_{9}=-\frac{1}{19683}. \end{array}$$

Furthermore, the characteristic polynomial of $\rho_{AB}^{T_{A}}$ is given by

$$\begin{array}{ccc} f(x) & = & x^{9}-x^{8}+\frac{8x^{6}}{27}-\frac{2x^{5}}{27}-\frac{2x^{4}}{81}+\frac{8x^{3}}{729}-\frac{x}{2187}+\frac{1}{19683}\\ & = & \frac{{\left(3x-1\right)}^{6}{\left(3x+1\right)}^{3}}{19683}. \end{array}$$

Thus, we get that

$$x_{1}=\cdots =x_{6}=\frac{1}{3},x_{7}=x_{8}=x_{9}=-\frac{1}{3}.$$

Therefore $\lambda_{\min}\left(\rho_{AB}^{T_{A}}\right)=-\frac{1}{3}$. □

From the above result, when n→∞, $\lambda_{\min}\left(\rho_{AB}^{T_{A}}\right)\to 0$ for a maximally entangled state in $C^{n}⊗C^{n}$. Based on the observation, we can conclude that the family of bipartite states with the minimal eigenvalue of their partial-transposed states ($-\frac{1}{2}$) is different from the set of maximally entangled states when the dimensions of the underlying spaces are larger than two. This also indicates that the magnitude of the only lowest negative eigenvalue of the partial-transposed state in higher-dimensional space would not be enough to identify the maximally entangled state when there is more than one negative eigenvalue. In fact, it is known that for higher dimensions the characterization of entanglement can be given by the so-called “negativity” [12], which is defined as the absolute value of the sum of all the negative eigenvalues of the partial-transposed state. That is, the negativity of $\rho_{AB}$ is given by $N\left(\rho_{AB}\right)=\left|\sum_{i}\min\left\{\lambda_{i}\left(\rho_{AB}^{T_{A}}\right),0\right\}\right|$. With this notion, our Theorem 1 can be rewritten: For the two-qubit state $\rho_{AB}$, $N\left(\rho_{AB}\right)=\frac{1}{2}$ iff $\rho_{AB}$ is maximally entangled. The success of such characterization lies in the possible number of negative eigenvalues being at most one. The reason for the failure of this result in high-dimensional space is that only one negative eigenvalue (the lowest one) would not be enough to characterize entanglement when there could be more than one negative eigenvalue.

## 3. Concluding Remarks

In this short note, we make an attempt to study the structure of a family of bipartite states with the extreme eigenvalue being $-\frac{1}{2}$ of its partial-transposed states. To characterize the maximally entangled two-qubit states, we employed the approach recently used by Yu et al. to study PT-moments, i.e., PT-moment vectors. In higher dimensional system, we were curious whether the PT-moment vector ($p^{\left(n^{2}\right)}=\left(p_{1},\ldots ,p_{n^{2}}\right)$ where $p_{k}=\frac{\left(n+1\right)+\left(n-1\right){\left(-1\right)}^{k}}{2n^{k-1}}$) generated by the maximally entangled states, only corresponded to the maximally entangled states. Clearly, a bipartite state in $D\left(C^{n}⊗C^{n}\right)$ with $\lambda_{\min}\left(\rho_{AB}^{T_{A}}\right)=-\frac{1}{2}$ was, in general, not maximally entangled unless n=2. In future research, we will continue to figure out the structure of this family of states, especially to find out the connection between it and maximally entangled states in a higher dimension. Furthermore, we will study the connection between the entanglement in bipartite states and the number of negative eigenvalues of the corresponding partial-transposed states.
